# Malingering and PTSD: Detecting malingering and war related PTSD by Miller Forensic Assessment of Symptoms Test (M-FAST)

**DOI:** 10.1186/1471-244X-13-154

**Published:** 2013-05-29

**Authors:** Khodabakhsh Ahmadi, Zeynab Lashani, Mohammad Hassan Afzali, S Abbas Tavalaie, Jafar Mirzaee

**Affiliations:** 1 Behavioral Sciences Research Center, Baqiyatallah University of Medical Sciences, P.O. Box: 19395–5487, Tehran, Iran; 2 Department of Psychiatry department, Baqiyatallah Hospital, Tehran, Iran; 3 Sadr Hospital, Tehran, Iran

**Keywords:** Post-traumatic stress disorder (PTSD), Miller forensic assessment of symptoms test (M-Fast), Malingering, Combat disorder

## Abstract

**Background:**

Malingering is prevalent in PTSD, especially in delayed-onset PTSD. Despite the attempts to detect it, indicators, tools and methods to accurately detect malingering need extensive scientific and clinical research. Therefore, this study was designed to validate a tool that can detect malingering of war-related PTSD by Miller Forensic Assessment of Symptoms Test (M-FAST).

**Methods:**

In this blind clinical diagnosis study, one hundred and twenty veterans referred to War Related PTSD Diagnosis Committee in Iran in 2011 were enrolled. In the first step, the clients received Psychiatry diagnosis and were divided into two groups based on the DSM-IV-TR, and in the second step, the participants completed M-FAST.

**Results:**

The *t*-test score within two groups by M-FAST Scale showed a significant difference (t = 14.058, P < 0.0001), and 92% of malingering war-related PTSD participants scored more than 6 and %87 of PTSD group scored less than 6 in M-FAST Scale.

**Conclusions:**

M-FAST showed a significant difference between war-related PTSD and malingering participants. The ≥6 score cutoff was suggested by M-FAST to detect malingering of war-related PTSD.

## Background

Post-traumatic stress disorder (PTSD) is one of the most prevalent psychiatric disorders in veterans [1]. PTSD was reported to be more frequent in Iranian warfare victims in comparison with other disabled veterans [1,2]. Malingering sometimes occurs among patients who seek treatment for PTSD as a result of war-related trauma experiences [3]. Pseudo-posttraumatic stress disorder refers to cases in which a patient’s presentation is a simulation of the actual clinical syndrome. Malingering of PTSD has been neglected by many clinicians and researchers who often rely on the assumption that a patient’s reported symptoms can be accepted as valid [4]. PTSD is particularly vulnerable to malingering because the diagnostic process heavily relies on the patient’s subjective report of his/her symptoms [5] and different interpretations on traumatic experiences and the severity of an individual’s emotional consequence make PTSD easy to malinger [6]. In addition, symptom overlap (insomnia, numbing, startle, anxiety, depression, fatigue, loss of affinity with others, etc.) makes the diagnosis even more difficult to determine [3,7]. In such studies, PTSD malingering is estimated to occur in at least 20% of compensation-seeking combat veterans [8]. Moreover, it is important for the assessor psychiatrist to understand his/her role in evaluating a patient claiming PTSD [5]. In a treatment or consultation setting, most therapists take their patients’ reported symptoms at face value since it is assumed that it is in the patients’ best interest to be honest with their doctors for the best treatment [5]. However, psychological testing can be useful in alerting the interviewer to possible malingering and to support the decision on malingering [5,9].

Psychological tests created and used to diagnose PTSD cannot detect malingering [10]. Lange et al. [9] showed that malingered PTSD cases had significantly higher scores in the majority of MMPI-2 and PAI validity indicators. Nelson et al. [11] suggested that among many MMPI-2 validity scales examined (e.g., L, F, K, Fb, Fp, and FBS), FBS yielded the largest effect size for differences between a group of participants with secondary gain and a group with no secondary gain. Another screening instrument for malingering of mental illnesses has recently been developed by Miller et al. [12] as an abbreviated version of the Structured Interview of Reported Symptoms (SIRS), the M-FAST. The M-FAST is a brief, structured interview that assesses malingering of psychotic symptoms based on the overall response style. Initial studies have found that the M-FAST is successful in detecting malingering in known group comparisons and it is moderately a useful assessment in classifying individuals as either honest responders or malingerers [13]. The outpatients’ disability claimants that were suspected of malingering had significantly higher total M-FAST scores than honest responders [12]. Strunk et al. [14] showed that M-FAST correctly identified 78% of coached malingerers. Guriel et al. [14] showed that M-FAST and Trauma Symptom Inventory (TSI) identified 90% cases of malingering of PTSD and Guy et al. [15] showed that malingers got more score than clinical participants in M-FAST (with Schizophrenia, major depression, bipolar and acute traumatic stress disorders). Furthermore, Jackson and colleagues [16] indicated that the ≥6 score in forensic assessment is a cutoff point for detected malingers in prisons. Although detecting war-related malingering is really important to clinicians, there are few published methods specifically designed for detecting exaggeration of psychological symptoms [7,17] and the structured inventory of malingering symptomatology across the world [7,18] and especially after Iraq-Iran war, in Iran. Therefore, this study was designed to detect malingering from war-related PTSD by Miller Forensic Assessment of Symptoms Test (M-FAST).

## Methods

In this blind clinical diagnosis study, the participants were 120 veterans referred to War Related PTSD Diagnosis Committee in Baqiyatallah and Sadr hospitals, in Tehran, Iran in 2011. All the participants were diagnosed as having PTSD of direct combat conditions, with none of them having PTSD of other related events (captivity, accidents, etc.). The addicted clients and the ones with organic illnesses were excluded from the study. This research had two steps. At the first step, the clients were visited by psychiatrists and evaluated for PTSD and finally were divided into two groups: some of them were diagnosed as PTSD, and for the second group, a PTSD diagnosis was ruled out. In this step, the diagnosis criteria were based on the Diagnostic and Statistical Manual of Mental Disorders (DSM-IV- TR, 2000). So, another research team performed the second step without knowing the results of the first team diagnosis. In this step, participants completed the Miller Forensic Assessment of Symptoms Test (M-FAST) for detecting malingering. In this study, we administered Persian version of M-FAST with acceptable internal consistency (α-Chronbach’s = 0.86). Finally the original researchers analyzed and compared the data of two research teams. The proposal of this research was approved by ethics committee in Baqiyatallah University of Medical Sciences. So, all of the participants voluntarily completed the consent form before entering the study. There was no coercion upon the study subjects for participation.

The brief structured interview of M-FAST includes 25 items and is designed to detect malingering by assessing individuals’ general response style. The M-FAST contains seven subscale scores and an overall total score. The seven subscales are based on strategies used to detect malingerers, reported versus observed symptoms, extreme symptoms, rare combinations, unusual hallucinations, unusual symptom course, negative image and suggestibility (Miller et al. 2000 & Miller, 2001). The re-test reliability for the total score over a one week interval was 0.92 among psychiatric inpatients, and alpha for the total score was 0.93 among inpatients and 0.92 in non-clinical participants (Miller, 2001). Miller (2001) recommends a cutoff score of 6 or greater to predict malingering, which showed a sensitivity of 0.93 and a specificity of 0.83 in a clinical population in an initial validity study.

The data were analyzed using descriptive methods, and the cutoff points were calculated by sensitivity, specificity, hit rate, and *t*-test. In this study, the diagnosis, response measurements and research on malingering war-related PTSD were conducted by three separate collaborating groups.

## Results

Baseline characteristics of the participants are demonstrated in Table 1. All participants were men and married with mean age of 45.6 in range of 445.93 to 45.63 years. The majority of participants were high school graduates.

**Table 1:** Baseline characteristics of malingering and PTSD groups

	**PTSD (n = 76)**	**Malingering (n = 44)**
Age (years)	47.18 ± 6.10	44.30 ± 4.80
Marital status, n (%)		
*married*	76 (100)	44 (100)
Level of education, n (%)		
*<high school*	19 (25.0)	10 (22.7)
*high school*	40 (52.6)	25 (56.8)
*Bachelor’s*	16 (21.1)	8 (18.2)
*Master’s and higher*	1 (1.3)	1 (2.3)

Mean, standard division and *t*-test for two groups (War-related PTSD & malingering for war-related PTSD) in M-FAST are shown in Table 2. According to the *t*-test score, (t = 14.058, df =116.31, P > 0.000) M-FAST shows a significant difference between war-related PTSD group and malingering group (Table 2). According to the M-FAST results (PTSD = 3.13 & Malingering = 8.28), PTSD patients had scored less than malingering participants. The comprehension of frequencies in two groups and predictive power values for M-FAST validity indicators are used to detect malingered PTSD and war-related PTSD (Table 2).

**Table 2:** **The mean, standard division, error’s standard division and *t*-test of two groups**

**Group**		**mean**	**Standard deviation**	**Minimum score**	**Maximum score**	**t-statistic**	**df**	**P value**
M-FAST	*Malingered*	8.28	2.12	2	13	14.05	116.31	0.0001
	*War PTSD*	3.13	1.88	1	7			

Abbreviation: *M-FAST*, Miller-Forensic Assessment of Symptoms Test; *df* : degree of freedom.

**Figure 1:**
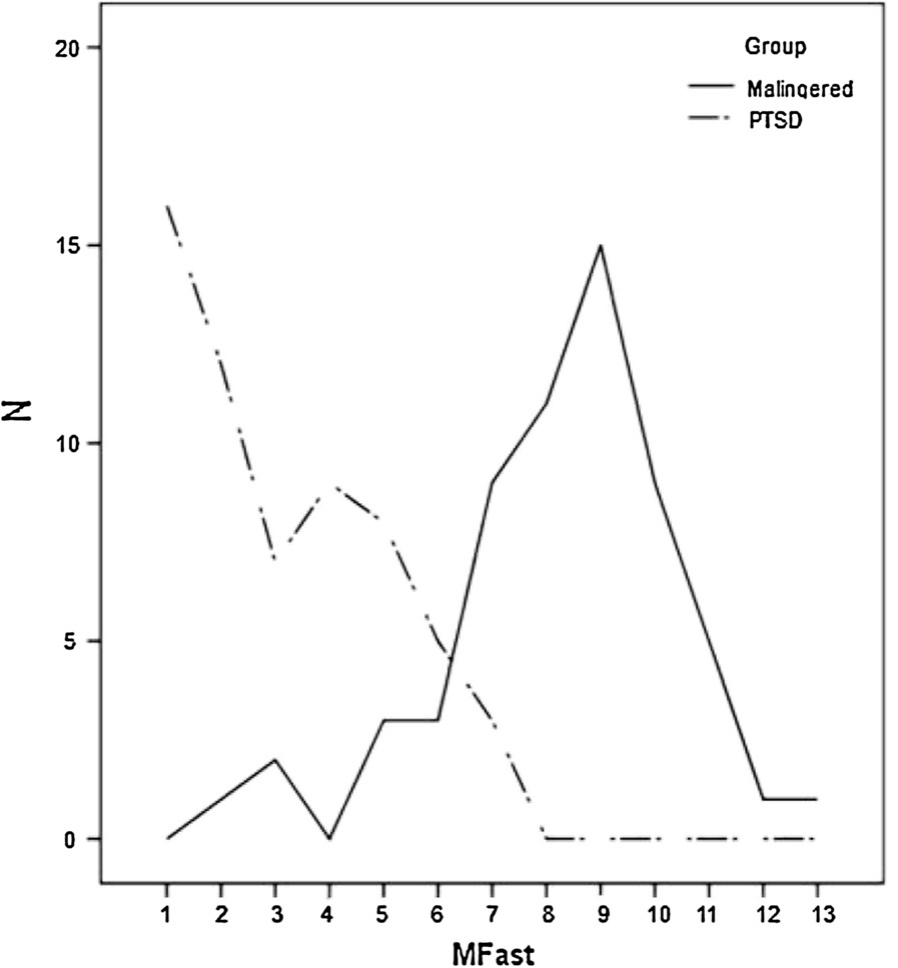
Plot of M-FAST by two groups.

According to Table 3, based on specificities (87) and sensitivities (92), the cutoff point 6 for M-FAST scales was the point of malingering group to war-related PTSD disorder. Also, 92% of malingers to war-related PTSD got more than 6 scores and %87 of PTSD group got less than 6 scores in M-FAST scale (Table 3). The results are shown in Figure 1.

**Table 3:** Operating characteristics of M-FAST scale for two groups

		**Malingering group**	**War PTSD group**	**Specify**	**Sensitivity**	**HR**
M-FAST	1	0	16	100	27	37
	2	1	12	100	47	27
	3	2	7	98	58	21
	4	0	9	95	73	16
	5	3	8	95	87	9
	6^a^	3^a^	5^a^	87^a^	92^a^	8^a^
	7	9	3	85	100	8
	8	11	0	70	100	15
	9	15	0	52	100	24
	10	9	0	27	100	37
	11	5	0	12	100	44
	12	1	0	3	100	48
	13	1	0	2	100	49
Total		60	60			

^a^: cutoff point; sensitivity: percentage of malingering and war-related PTSD groups correctly classified at cutting score; specificity: percentage of malingering and war-related PTSD groups correctly classified at cutting score; HR, overall hit rate: percentage of all groups correctly classified.

### Note

Sensitivity is the true positive (Hit) rate for the test (number of people with positive test result divided by summation of true positives and false negatives). Specificity is the true negative rate (number of people with true negatives and false positives). Sensitivity and specificity are directly dependent on the classification scheme (cut off) employed with a given diagnostic technique and are independent of base rates (Hennekens & Buring, 1987). For clinical diagnosis, sensitivity and specificity must be “translated” into positive predictive power (PPP) and negative predictive power (NPP) which are dependent on base rates (Gouvier, Hayes & Smiroldo, 1998).

## Discussion

In the present study, we found that there is a significant difference between the two groups regarding diagnosis of PTSD. According to the *t*-test score (t = 14.058, df =116.31, P < 0.001) within the two groups by M-FAST scale, there was a significant difference between war-related PTSD and the malingering group. Based on specificities (87) and sensitivities (92), the cutoff point of 6 in M-FAST scales was the point of malingering group to war-related PTSD disorder. Also, 92% of malingering of war-related PTSD people scored more than 6 and %87 of PTSD group scored less than 6 in M-FAST scale. Whereas the prevalence rates or the percentage of individuals with PTSD are obvious in a lifetime after wars, it seems necessary to evaluate veterans for PTSD every year. Accordingly, the M-Fast can be suitable for detecting the malingering of PTSD.

These finding are the same as those in previous studies, for example: Strunk et al. [14] showed M-Fast correctly identified 78% of coached malingerers. Guriel et al. [19] showed M-FAST and TSI detected 90% of malingering to the PTSD and Guy et al. [15] suggested that malingering people scored higher in the M-FAST than clinical participants (with Schizophrenia, major depression, bipolar and acute traumatic stress disorders). Jackson et al. [16] indicated that the ≥6 score is a cutoff point for detecting malingering people in forensic assessment in prisons.

So, our study is in accordance with the previous ones, and shows that M-FAST scales can be effectively used in Iranian veterans with PTSD symptoms. This probably proves that cultural aspects play no major role in the validity of M-FAST scale to detect malingering in veterans. However, one should consider that despite the high detection rate of malingering achieved by M-FAST, it is not 100% diagnostic, and still a good proportion of undetected cases exist. So, interpretation of findings derived from M-FAST should be undertaken cautiously.

This study has some powerful points as well as limitations. As a strength point for this study, we can note the relatively large number of the study subjects, and the data from Iranian veterans of Iraq-Iran war. Moreover, we used a blind assessment to address the PTSD in the current study. We also used standardized measures in our approach. On the other hand, we can call the long time after the war as a weak point for our study, while this might be able to adversely affect the validity of our study results.

## Conclusion

The present results suggest M-FAST for detecting malingering of war related PTSD cases among Iranian veterans because M-FAST is a brief scale with high rate of validity in this patient population. Of course, we need further studies for investigating M-FAST ability to detect malingering for other disorders.

## Competing interests

The authors declare that they have no competing interest. The authors specify that, we haven’t any financial competing interests (political, personal, religious, ideological, academic, intellectual, commercial or any other) to declare in relation to this manuscript.

## Authors’ contributions

KA conceived the study, participated in its design, coordination, and interpretation of the results. MHA was involved in recruiting patients and collection of data. ZL performed statistical analyses. JM contributed to patient recruitment and also prepared early draft of the manuscript. SAT participated in visiting patients and diagnosed PTSD. All authors read and approved the final manuscript.

## Pre-publication history

The pre-publication history for this paper can be accessed here:

http://www.biomedcentral.com/1471-244X/13/154/prepub
